# Association between Vitamin D Receptor Gene BsmI Polymorphism and Bone Mineral Density in A Population of 146 Iranian Women

**Published:** 2013-05-05

**Authors:** Farkhondeh Pouresmaeili, Javad Jamshidi, Eznollah Azargashb, Shahdokht Samangouee

**Affiliations:** 1 Department of Medical Genetics and Infertility and Reproductive Health Research Center (IRHRC), Faculty of Medicine, Shahid Beheshti University of Medical Sciences, Tehran, Iran; 2 Department of Biochemistry, Fasa University of Medical Sciences, Fasa, Iran; 3 Department of Medical Genetics, Faculty of Medicine, Shahid Beheshti University of Medical Sciences, Tehran, Iran; 4 Department of Social Medicine, Faculty of Medicine, Shahid Beheshti University of Medical Sciences, Tehran, Iran; 5 Romatology Clinic, Taleghani Hospital, Shahid Beheshti University of Medical Sciences, Tehran, Iran

**Keywords:** Vitamin D Receptor, BsmI, Polymorphism, Bone Mineral Density, Osteoporosis

## Abstract

**Objective:**

Osteoporosis is a bone disorder that reduces bone mineral density (BMD) and leads to bone fracture. In addition to different factors, gene polymorphisms have been revealed to be associated with osteoporosis. In this study, we investigated the association between the BsmI polymorphism of vitamin D receptor (VDR) gene (rs1544410) and BMD in a population of Iranian women.

**Materials and Methods:**

In this case control study, clinical risk factors for osteoporosis were obtained from the participants through a questionnaire for a case-control study. The World Health Organisation (WHO) criteria were applied for the diagnosis of the disease. Peripheral blood samples were obtained from 146 pre- and or postmenopausal Iranian women aged between 35 and 71 years (53.53 ± 9.8). The study population was classified for BMD into normal and osteoporotic groups, who matched for age, pregnancy status, menstrual condition, and body mass index (BMI). The BMD of the lumbar spine (L1-4) and femoral neck was measured. Polymerase chain reactionrestriction fragment length polymorphism (PCR-RFLP) was performed to detect and analyze the genotype.

**Results:**

The frequencies of AA and GG were significantly different between the two groups (p value<0.05), with the first genotype being higher in the patients and the second being higher in the normal group. The GG genotype was significantly associated with increased BMD in the lumbar spine (p value<0.05) but non-significant in the femoral neck (p value>0.05).

**Conclusion:**

BsmI polymorphism of VDR gene has a significant association with BMD in the lumbar spine and may have a minor effect on the proximal femur BMD in Iranian women.

## Introduction

Osteoporosis is a multifactorial disease common inpostmenopausal women and related to age. It is characterized by bone mineral density (BMD) decline, elevated risk of bone fractures, and skelet aldisturbance (1). There are many factors associated with the disease such as gender, age, body mass index (BMI), nutrition, menopause status and even number and age of pregnancies (2-6). Osteoporosis is induced by environmental as well as genetic factors. Genetic factors are thought to account for approximately 50-80% of inter-individual BMD variability (7, 8).

Vitamin D receptor (VDR) gene is localized on 12q12-14 and several of its polymorphisms have been reported (9). This gene is a combination of 11 exons and is approximately 75 kb in length. The 5' UTR region of the VDR gene is composed of three exonic sequences called 1A, 1B, and 1C, while its translated product is encoded by the other eightexons (10). The large promoter region of the gene enables it to produce different tissue-specific transcripts (11).

Vitamin D active form, which can interact with VDR, is 1, 25-dihydroxyvitamin D3 (1, 25 [OH] 2D3, or calcitriol) and is the hydroxylated metabolite of vitamin D3 (12, 13). Vitamin D (1, 25-Dihydroxyvitamine D3) is involved in bone metabolism and is recognized to be an inducer for bone synthesis through binding to its receptor (VDR), resulting in skeletal cell stimulation and bone turnover regulation (14). According to its functions, VDR gene seems to be involved in the genetic determination of bone mineral density and osteoporosis.

There are numerous conflicting studies on some gene polymorphisms of VDR to investigate their association with BMD and their potential roles in the susceptibility to osteoporosis. Most of the studies and linkage analyses have identified three adjacent restriction fragment length polymorphisms for BsmI, ApaI, and TaqI in VDR gene (11, 15, 16). The functional effects of some of VDR polymorphisms are known and they include changes in the receptor binding affinity (11, 17). Since the BsmI polymorphism site is located in close proximity to the 3’ untranslated area, it is hypothesized that the altered form of the receptor gene could influence the stability of its transcript (11). However, there is a knowledge gap for the mechanism of the BsmI polymorphism function and its probable effects on the gene transcript and/or protein structure. In a study on VDR BsmI polymorphism , Houston et al. (18) realized that individuals with the AA genotype had a higher rate for femoral neck bone density than individuals with the GG genotype. An inverse finding was reported by Morrison et al. (10).

Given the controversy in the existing association reports, it is advisable that these associations be evaluated in different populations. This polymorphism is the most common VDR polymorphism studied so far and has shown association with BMD variations. In the current study, we have also investigated the association between the VDR gene BsmI polymorphism and BMD in a population of pre- and post menopausal Iranian women for the first time. The results of this study can helppredictosteoporosis risk in Iranian women.

## Materials and Methods

### Study population

Patient information was obtained with a questionnaire. Women referred to the Rheumatology Clinic and BMD Department of Loghman Hospital in Tehran, Iran, due to acute skeletal pain underwent Dual Energy X-Ray Absorptiometry (DXA). The number of patients was statistically calculated using a comparison of two proportions with α=5% and β=10%. Of 146 unrelated randomly selected women, 64 individuals met the criteria of osteoporosis according to their BMD values and World Health Organization (WHO) criteria (19), and 82 women did not meet the criteria for being osteoporotic. A detailed profile, including medical, personal, and family history was obtained from all the subjects who were aged between 35 and 71 years (mean of 53.53 ± 9.8 years) and both normal and patient groups were matched for menstrual condition, age, height, weight, pregnancy rate and occupation.

Women with acute pain, physical examination, X-ray absorptiometry, and BMD values between -1 and -2.5 were recruited in this study as patients /controls. Those with a history of using hormonal medications or calcium supplement tablets or those with any dietary habits that would affect bone mass and turnover were excluded from the study.

All the patients received comprehensive explanation about the aim of the research and thereafter signed a consent form. The research was a case-control study, and it was approved by Shahid Beheshti University of Medical Sciences’ Research Council (No.1147) and confirmed by the University’s Ethics Committee.

### BMD measurement

BMD (g/cm2) at lumbar spine (L1-4) and femoral neck was measured for each subject using DXA (DXA; Lunar DPXL Densitometer, Lunar Corp., Madison, WI).

### Genotyping

Genomic DNA was extracted and purified from EDTA blood samples taken from each volunteer using the method of Miller et al. (20). Genotyping analysis of VDR gene BsmI polymorphism (rs1544410) was performed by Polymerase Chain Reaction Restriction Fragment Length Polymorphism (PCR-RFLP). The primers were designed to amplify a 191bp fragment, including a restriction site located in intron 8 of the gene.

### Polymerase chain reaction

Amplification of a 191-bp genomic fragment was performed using 100-200 ng of the extracted DNA in 2.5 µl of buffer solution [1X PCR buffer (50 m MKCl; 10 m MTris-HCl; 1.5 m M MgCl2), 2 m M MgCl2, 200 µM dNTP mix and DDW was added up to 25 µl] with 1 unit of TaqI DNA polymerase (super Taq DNA polymerase, Gen Fanavaran Co., Tehran, Iran) and 0.4 µM of each oligonucleotide primer (Forward primer: 5'AGTGTGCAGGCGATTCGTAG3', Reverse primer: 5'ATAGGCAGAACCATCTCTCAG3').

**Fig 1:**
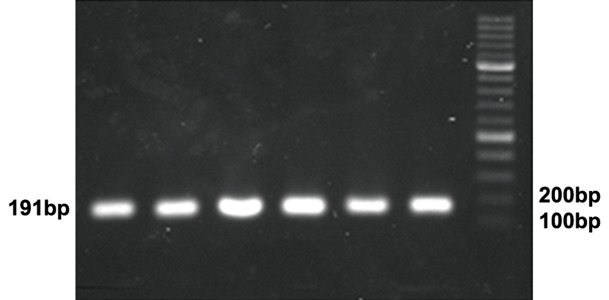
Amplification of VDR gene fragment. PCR products are shown on 1% Agarose gel. The marker is a 100bp ladder

PCR was performed for 35 cycles with the following steps: denaturation at 94˚C for 4 minutes; 35 cycles of denaturing at 94˚C for 30 seconds; annealing at 58.5˚C for 30 seconds; and extension at 72˚C for 25 seconds with a final extension of 5 minutes at 72˚C. The PCR products were loaded onto a 1% Agarose gel with ethidium bromide staining. The length of the product was confirmed to be 191 bp (Fig 1).

### Restriction digest

**Fig 2:**
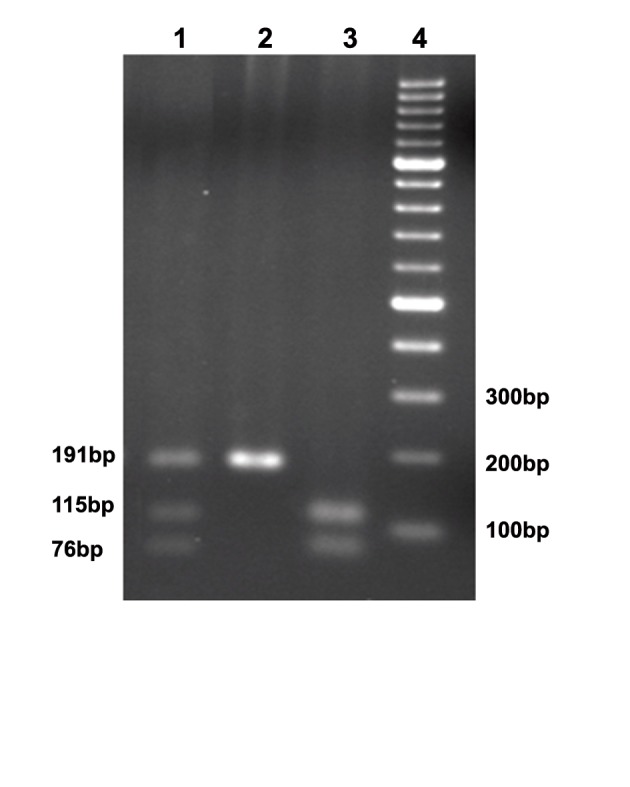
Restrictions digest of PCR product. The first well shows the pattern of heterozygote (A/G), the second well shows the pattern of homozygote for A/A, and the third well shows the pattern of homozygote for G/G. The fourth well is a 100bp DNA ladder

After amplification of the target DNA, 5µl of each PCR product was digested with 1 unit of BsmI restriction enzyme (Vivantis RE1310, City, Country) at 37˚C for about 2 hours. BsmI cuts the GAATG^C sequence between the G and C, as is shown here by the "^" symbol. When there is a guanine (G) in this region, BsmI cuts the DNA. In contrast, when the G is converted to adenine (A) after a SNP (rs1544410) event, the enzyme cannot recognize its restriction site and does not cut the DNA. Therefore, the enzyme digestion results in the production of two fragments in different lengths of 115bp and 76bp. The digested PCR products were analyzed on 2% Agarose gel stained by ethidium bromide (Fig 2).

### Statistical Analysis

All the statistical analyses were carried out using the SPSS software package (SPSS 16.0.0, Chicago, IL, USA). In this manuscript, the results are presented as mean ± standard deviation. Also, changes in BMD were analyzed by non-paired T-scores and Z-scores. The genotype frequencies of the controls and patients were compared using the Pearson’s chi-square and Fisher’s exact tests. A p value <0.05 was considered statistically significant

## Results

The study subjects were unrelated and aged between 35 t0 71 years with an average spine BMD of -1.296 (Z-score) and -1.959 (T-score) (Table 1). As is shown in Table 1, the mean of spine BMD in the control group was 0.41 by Z-score and 0.285 by T-score. The difference between the two groups in spine BMD according to T-scores and Z-scores was significant (p value ≤0.0001) (14).

Polymerase chain reaction amplified an expected region of 191 base pairs of VDR gene (Fig 1). Consequent restriction digest by BsmI resulted in two bands of 76 bp and 115 bp in homozygotes for the restriction site, three different fragments of 191 bp, 76 bp and 115 bp in heterozygotes for BsmI restriction sequence, and one single 191-bp fragment in the samples which were homozygote with no restriction site (Fig 2).

The frequency of A/G alleles and the related genotypes was identified and confirmed in the study population. The genotype frequency was measured for all of the participants. From the total affected people, 14 individuals demonstrated AA genotype (~22%), while 17 patients had genotype GG (~27%). In contrast, of a total 82 normal people, 13 had AA (~16%) and 36 demonstrated GG genotype (~44%) (Table 2). In addition to the data, allele A and G frequencies were measured in the population under study, where allele G showed an increase level in the normal group (p value<0.05). It seemed that the GG genotype was positively associated with BMD increase in the lumbar spine but showed no significance in the femoral neck BMD rate.

**Table 1:** Comparison between spine and femur bone mass density mean values in the case and control groups according to Z and T scores groups


Groups	(Mean ± SD)

**SP.Z Control Case**	0.411 ± 1.0* -1.296 ± 0.843
**SP.T Control Case**	0.285 ± 1.1 -1.959 ± 0.887
**SP.BMD Control Case**	1103.33 ± 124.281 846.46 ± 100.483
**FEM.Z Control Case**	0.741 ± 0.919 0.393 ± 0.873
**FEM.T Control Case**	0.700 ± 1.146 -0.862 ± 1.02
**FEM.BMD Control Case**	967.26 ± 112.713 797.53 ± 119.27


SP.Z; Spine Z-score, SP.T; Spine T-score, SP.BMD; Spinebone density, FEM.Z;Femur Z-score, FEM.T; Femur T-score and FEM.BMD; Femur bone density. *P value <0.001.

**Table 2:** Comparison between A and G Genotypes and allele frequencies between the case and control groups


Sample	All	AA	AG	GG	A	G

**Total**	146	27(18.4%)	66(45.8%)	53(36.3%)	41%	58.9%
**Affected**	64	14 (21.8%)	33 (51.5%)	17 (26.5%)	47.6%	52.3%
**Normal**	82	13 (15.8%)	33 (40.2%)	36 (43.9%)	35.9%	64%


## Discussion

Gene polymorphism has been one of the most discussed genomic variations in the recent years. The significance of these variations is well known in the early diagnosis of genetic diseases and economic effects on public health (7-9). Osteoporosis is one of the well-recognized bone diseases and affects millions of people, especially women, worldwide each year (1). Osteoporosis has a polygenic nature (21) and therefore, complicates early diagnosis programs.

Sincevitamin D receptor is expressed in different tissues like muscle, bone, skin, and gonads, it is expected to have biological effects on growth, reproduction and other body systems (22). The association between vitamin D receptor BsmI polymorphism and BMD has been demonstrated in a great number of studies (7, 23-25) and is confirmed by our present research, where we assessed the presence and association of BsmI polymorphism in a group of normal and osteoporotic Iranian women through a case-control study. Be that as it may, some investigations have found no relation between VDR gene polymorphisms and BMD in different populations (26, 27). Suh et al. (28) suggested a significant association between VDR BsmI polymorphism and lumbar spine BMD in affected Korean girls with adolescent idiopathic scoliosis. The controversies between different studies could be attributed to numerous reasons, first and foremost among which is the positive environmental influence on bone mass (29-31).

It is understood that age-related bone loss is asymptomatic and the genotypes could influence differently on BMD in various locations of the skeleton (32). In our study, GG genotype was significantly associated with increased BMD in the lumbar spine. Houston et al. (18) found that individuals with AA genotype had a higher femoral neck bone density than individuals with GG genotype. The frequencies of AA, AG and GG vary in different populations: the frequencies in Chinese women were 2.3, 18.1, and 79.6%, while the Caucasian population showed frequencies of 15.4, 47.4, and 37.2% for the same genotypes (16, 33). The reason for such inconsistencies between these studies can be attributed to various factors such as the sample size, study design, age, ethnic ancestry, and lifestyle factors (physical activity, obesity, calcium intake), all of which could affect gene regulation in different genotypes and subjects and result in BMD loss or gain (3, 7).

Several studies have demonstrated a statistically significant higher BMD in VDRBsmI heterozygote subjects in the Chinese population (17, 23).In contrast, the evaluated groups in our study showed to be either homozygote or heterozygote for the polymorphism, but there was a significantly higher BMD in the homozygote Iranian population. The frequency of AA genotype was higher in the patients and GG genotype was higher in the normal group, which could explain the importance of G allele existence in the normal bone mass structure. It has been reported that this functional polymorphism of intron 8 affects the receptor gene expression (34) and, as was previously mentioned, this may be through mRNA instability (11). It has also been revealed that VDR polymorphisms alter the circulation of vitamin D (35). However, there is no evidence to show how the gene variant would change the gene regulation or its transcript or protein structure, and nor is there any evidence to demonstrate by which mechanism it would play a role in vitamin D intake by living cells. To understand the exact role of the polymorphism, it is necessary not only to compare the entire genome of the people of the same genotype either healthy or patient, but also toanalyze the genomic organization of the VDR locus and to identify the relationship between the genes present in the same chromosomal area and to identify other possible gene-gene interactions throughout the whole genome to be able to analyze the association mechanism of the polymorphism with a phenotype like osteoporosis.

The present study has some limitations, the most notable of which was that the subjects in the two groups were not totally matched, for example in terms of lifestyle. However, the results of this research suggest that G allele has no dominant effect onA allele; but in homozygote status, GG genotype is highly related to an increased level of BMD in Iranian women.Due to the possible presence of other unknown genetic or non-genetic factors which could be in interaction with the polymorphism, patients and normal individuals carrying the same genotype may express different health conditions.

## Conclusion

In our studied population, BsmI G>A gene polymorphism at the VDR locus played a significant role in the etiology of osteoporosis; this was confirmed by a positive loss of BMD in the patients. Therefore, we conclude that this genetic variant could be a possible genetic marker of BMD in Iranian women susceptible to the disease.

Nonetheless, the result of this study can only be validated after examination of a larger population with the exact genotype influence of this type on BMD. The results of future investigations can be used to understand differential therapy responses based on patient’s VDR genotype.

A significant association between the gene polymorphism and osteoporosis related to VDR gene could be a valuable biological tool not only for the early diagnosis of osteoporosis and its therapy but also for the prediction of susceptibility in women at high risk forosteoporosis.
